# Association between early enteral nutrition and mortality in critically ill patients

**DOI:** 10.1186/s13054-023-04697-y

**Published:** 2023-10-23

**Authors:** Shangzhong Chen, Caibao Hu

**Affiliations:** Department of Intensive Care, Zhejiang Hospital, 1229 Gudun Road, Hangzhou, 310013 Zhejiang China

To the Editor,

A recent study [1] investigated the association between early nutrition support and 28-day mortality in critically ill patients. This study was conducted in 26 ICUs, and a total of 1206 patients were included. The authors reported that in critically ill patients, early nutrition support was significantly associated with increased 28-day mortality. This conclusion is different from previous studies. Several factors may cause these different findings.

Early nutrition support plays a key role in critically ill patients. According to the ESICM guidelines [2, 3], early nutrition support (within 48 h) in critically ill adult patients should be performed unless the presence of relative contraindications, such as uncontrolled shock, uncontrolled hypoxemia and acidosis, uncontrolled upper gastrointestinal bleeding, abdominal compartment syndrome, etc. Thus, in previous studies, patients who received early nutritional support were more likely to have mild disease severity, or at least no worse than those who received late nutrition support. For instance, one study [4], including 1353 neurocritically ill patients, reported that early enteral nutrition reduced the risk of in-hospital mortality and infectious complications. In this study [4], early nutrition support was defined as enteral or parenteral nutrition within 72 h after ICU admission. Compared to the early nutrition support group, the APACHE II score on ICU admission (8.3 ± 7.7 vs. 6.13 ± 5.5, *p *< 0.001), the proportion of patients receiving mechanical ventilation (67.3% vs. 53.9%, *p *< 0.001) and vasopressors use (16.5% vs. 11.7%, *p *= 0.033) were significantly higher in the late nutrition support group. Similarly, another retrospective study [5] conducted in critically ill patients also reported that early nutrition support reduced ICU mortality. In the baseline comparisons, both the proportion of patients receiving mechanical ventilation (91% vs. 71%, *p *= 0.01) was significantly higher in the delayed nutritional support group and the APACHE II score (18.5 ± 7.3 vs. 19.1 ± 8.8, *p *= 0.70) was comparable between two groups. In addition, in a study [6] with comparable disease severity status (comparable SOFA score or vasopressors use proportion), Reignier et al. reported that in 3032 patients with invasive mechanical ventilation > 72 h and shock, early nutrition was associated with lower day-28 mortality.

However, in the current study, we noted that compared to the “no early nutrition” group, the SOFA score (7.0 [4.0, 10.0] vs. 9.0 [6.0, 11.0] vs. 8.0 [4.0, 11.0], *p *< 0.001) or SAPS II score (41.0 [31.0, 53.0] vs. 47.0 [36.0, 60.0] vs. 44.0 [32.5, 57.0], *p *< 0.001) on admission was significantly higher in the early enteral or early parenteral groups. The proportion of patients receiving vasopressor use (49.8% vs. 75.6% vs. 61.2%, *p *< 0.001) or invasive mechanical ventilation (53.1% vs. 92.8% vs. 67.8%, *p *< 0.001) was also significantly higher in the early enteral or early parenteral groups. Thus, different from previous studies, early nutrition support was more likely to be prescribed in patients with worse conditions in the current study. The selective use could generate a strong internal correlation between early nutrition support, worse conditions and higher mortality, which may affect or overwhelm the “true” association between early nutrition support and mortality even in adjusted analysis. In addition, confounding factors such as SAPS II score, vasopressor use, or invasive mechanical ventilation were not included in the propensity score analysis, which may also increase the risk of bias. More trials are needed to validate these findings.

## Data Availability

Not applicable.
